# Risk factors for surgery in children with ureteropelvic junction obstruction due to antenatally detected ınfantil hydronephrosis

**DOI:** 10.1007/s10157-025-02631-w

**Published:** 2025-01-31

**Authors:** Mehmet Baha Aytac, Sule Ayas Ergul, Kenan Dogan, Neslihan Dincer Malkoc, Merve Aktas Ozgur, Cuneyd Ozkurkcugil, Kerem Teke, Busra Yaprak Bayrak, Zelal Ekinci, Kenan Bek

**Affiliations:** 1 Department of Pediatric Nephrology, School of Medicine, Kocaeli University, Kocaeli, 41001 Türkiye; 2 Department of Pediatric Nephrology, Kocaeli City Hospital, Kocaeli, 41001 Türkiye; 3 Department of Urology, School of Medicine, Kocaeli University, Kocaeli, 41001 Türkiye; 4 Department of Pathology, School of Medicine, Kocaeli University, Kocaeli, 41001 Türkiye; 5 Department of Pediatric Nephrology, Istanbul Hospital, Baskent University, Istanbul, 34662 Türkiye

**Keywords:** Ureteropelvic junction, Pyeloplasty, Infant, Hydroneprosis, Histopathology

## Abstract

**Background:**

Although the majority of cases with antenatally detected hydronephrosis (ANH) resolve during postnatal period; patients should be monitored for the risk of developing ureteropelvic junction obstruction (UPJO) which requires surgical intervention. We aimed to define independent risk factors for operation in whom diagnosis of UPJO was precisely proven with histopathological evidence.

**Methods:**

Medical files of 155 children (186 renal units) with anteroposterior pelvic diameter (APPD) ≥ 7 mm or ≥ 1SFU (Society of Fetal Urology) grade of pelvicalyceal dilatation were retrospectively investigated. Patients who underwent pyeloplasty and whose pathological examinations of resected ureteral samples confirmed obstruction, were compared to non-interventional group in terms of demographics, serum creatinine, APPD, SFU grade, cortical thickness and diuretic renogram. Multiple regression models were used to predict independent risk factors for pyeloplasty.

**Results:**

155 patients (186 renal units) were recruited for the study. Pyeloplasty was performed in 50(32.2%) patients. Increased APPD, T1/2 and Tmax values with low parenchymal thickness and DRF were demonstrated in operated patients compared to those who did not. Significant decrease in APPD and T1/2 values and also significant improvement in parenchymal thickness were observed in conservatively managed group. Multivariate analysis revealed high APPD measurements and time activity curve patterns to be associated with significantly increased likelihood of surgical intervention.

**Conclusions:**

There is still an ongoing debate on which screening method should be used for the accurate diagnosis of UPJO or the indications for surgical intervention. Baseline APPD and diuretic renogram curve were found to be significant in predicting surgery for UPJO.

## Introduction

Dilatation of renal collecting systems is the most common congenital abnormality detected in prenatal or neonatal ultrasound imaging [1–3]. Although majority of them are transient and resolve spontaneously after birth; some might be clinically significant like ureteropelvic junction obstruction (UPJO) with the potential of progressive loss of renal function if not treated with optimal surgical intervention [3]. Contrary to the previous years, prompt surgical management immediately after diagnosis of UPJO, has now been replaced by strict serial monitoring of pelvicalyceal system dilatation and selective surgical approach to be performed after the detection of deteriorating differential renal functions (DRF) [4]. However, there is still a debate about the preferred methods to demonstrate the progression of hydronephrosis and worsening kidney functions at an early stage before permanent damage occurs and which patients will benefit from pyeloplasty [5]. Therefore, in this study, we aimed to describe the clinical course of patients with pelvicalyceal system dilatation during postnatal period and the risk factors predictive for surgery in patients with UPJO, where obstruction was confirmed intraoperatively and also postoperatively with histopathological examination of ureters.

## Materials and methods

We retrospectively evaluated the medical charts of all neonates and infants who were followed up by the department of pediatric nephrology at Kocaeli University between July 2004 and May 2023 and also whose urinary system ultrasounds had revealed unilateral or bilateral hydronephrosis either with detection of anteroposterior pelvic diameter (APPD) ≥ 7 mm or any degree of calyceal deformity. The records were reviewed for demographical data, presence of antenatal hydronephrosis (ANH), duration of follow-up, initial serum creatinine levels, estimated glomerular filtration rate (eGFR) and incidence of surgical intervention with pyeloplasty. eGFR (ml/dk/1.73m^2^) was calculated according to the method reported by Schwartz et al. [6]. First sonographic and nuclear renogram results were noted for all patients, and they were repeated just before the surgery for those who underwent pyeloplasty and at the last office control for those who had been monitored conservatively. Patients with urolithiasis, ureteral dilatation, vesicoureteral reflux (VUR), anatomic and neurogenic dysfunction of the lower urinary tract were excluded. The study protocol was approved by local ethics committee of the university.

Ultrasonographic evaluations of the patients were made by radiologists. APPD was defined as the maximum distance measured between anterior and posterior parenchymal lips at the level of renal hilus on a transverse section. The severity of calyceal dilatation were graded using the Society of Fetal Urology (SFU) classification [7]. Cortical thickness (CT) was measured at various renal poles and minimum CT was recorded. The difference in parenchymal thickness of the affected kidney compared to the normal side was defined as parenchymal thinning.

Radionuclide renography was undertaken in cases of severe hydronephrosis or cortical thinning on ultrasound. Mercaptoacetyltriglycine-3 (MAG-3) scan was performed in the supine position without parenteral hydration or urethral catheterization. Furosemide at a dose of 1 mg/kg was given intravenously 3 min after the administration of radionuclide. Time-activity curves, T1/2 (minute) and Tmax (minute) values were recorded to assess drainage properties of the hydronephrotic kidney after diuretic administration. Renogram curves were classified as obstructive, non-obstructive and intermediate patterns as defined by the Society for Nuclear Medicine [8].

Medical charts revealed that gradual increase of hydronephrosis, APPD or parenchymal thinning in repeated ultrasounds along with the scintigraphic detection of serious loss of renal functions or obstructive curves were used as indications for surgery.

Inflammatory cell infiltration, fibrosis, irregular muscle fiber arrangement and smooth muscle hypertrophy within the layers of removed ureteral segment were accepted as histopathological confirmation of UPJO [9–12].

Data were mentioned as mean ± standard deviation for continuous variables and median (interquartile ranges) for skewed ones. Chi square test and Mann–Whitney *U* test were used for analysis. Univariate (log rank test) and multivariate regression models in terms of APPD, SFU grade, parenchymal thickness of the initial ultrasound and split functions, time-activity curves, T1/2 and Tmax values of the first nuclear renograms and baseline serum creatinine levels were performed to predict the independent risk factors for pyeloplasty. SPSS 22 statistical software was used and *p* value of 0.05 or lower was considered significant.

## Results

### Study population

A total of 155 patients (186 renal units) were recruited for the study. 124 of them (80%) had unilateral, 31 (20%) had bilateral pelvicalyceal dilatation. Of the 124 cases with unilateral hydronephrosis, it was left-sided in 90 patients (72.6%) and right-sided in 34 (27.4%). There were 112 boys (72.3%) and 43 (27.7%) girls. ANH was determined in 124 (80%) of all patients.

### Initial assessments

The median age of initial ultrasound was 45 days (range 25–90 days). Median APPD and parenchymal thickness was measured as 13 mm (9–18) and 7 mm (4–9), respectively. Cortical thinning was detected in 58(31.2%) out of 186 renal units. Of the 186 renal units, 31 (16.7%) had SFU grade 1, 57 (30.6%) had grade 2, 68 (36.6%) had grade 3, 9 (4.8%) had grade 4 hydronephrosis. 21 (11.3%) renal units had no calyceal dilatation.

Diuretic MAG-3 scintigraphy was performed for 86 (55.5%) cases (112 renal units) at presentation on a median age of 0.25 years (0.21–0.75). DRF ranged from 47.25 to 53.87% (median 51). Median values of T1/2 and Tmax were 14.15 min (9.75–24.25) and 3 min (2–12.48). 52 (46.4%) out of 112 renal units revealed obstructive, 24 (21.4%) intermediate and 36 (32.1%) non-obstructive pattern on radionuclide scan. DRF < 40% was demonstrated in 12 (10.7%) renal units.

Basal serum creatinine level ranged from 0.26 mg/dl to 0.43 mg/dl (median 0.37) with a median eGFR of 73.31 ml/dk/1.73m^2^ (56.25–103.21).

### Last assessments

The average age for final ultrasound was between 1.5 and 5.58 years (median 3). Median APPD and parenchymal thickness were measured as 10 mm (6.65–17.5) and 8.5 mm (5–11), respectively. Of the 163 renal units underwent the ultrasonographic examination, 32 (19.6%) had cortical thinning. The final ultrasound showed normal caliceal structure in 83 (51%) renal units, SFU grade 1 in 20 (12.2%), grade 2 in 28 (17.1%), grade 3 in 31 (19%) and grade 4 hydronephrosis in 1 (0.7%) renal unit.

In 37 (23.9%) out of 155 patients (45 renal units), final MAG-3 scan was performed between the ages of 1 and 4.5 years (median 2). Average DRF, T1/2, Tmax values were 49% (46.8–53%), 12 min (8–32) and 3 min (2–9.5), respectively. Obstructive renogram curve was present in 24 (53.3%), intermediate in 5 (11.1%) and non-obstructive pattern in 16 (35.6%) of 45 renal units.

The age at last office visit was 4.25 years (range 2.33–7.16 years) and the median duration of follow-up for all patients was 3,91 years (2.16–6.75).

### Pyeloplasty vs. conservative management

During the course of conservative therapy, pyeloplasty for UPJO was needed for 50 (32.2%) cases (50 renal units). Surgical notes revealed that a dynamic and stenotic ureter segments were observed in all patients during pyeloplasty. Obstructive histopathological changes were also demonstrated in ureteral samples of all operated patients. This was the finding that indirectly proved the accuracy of our surgical indications. Median age for surgical intervention was 1 year (0.66–2.33) and it was predominantly performed on the left side (74%).

Of the 50 patients having surgical correction for UPJO, 34 (68%) of them were boys and 16 (32%) were girls, while there were 78 (74.2%) boys and 27 girls (25.8%) in the conservatively managed group. The prevalence of ANH was 76% in those who underwent pyeloplasty and 81.9% in those who did not.

No difference was found between the ages of initial ultrasound and MAG-3 scintigraphy, when 50 operated renal units were compared to non-operated 136 [median 54 days (30–90) vs. 40.5 days (17–85.5) *p* = 0.121 and median 0.33 years (0.25–0.91) vs. 0.25 years (0.16–0.5) *p* = 0.071, respectively]. Duration of follow-up was similar in both groups [median 3.41 years (1.87–5.97) vs. 4.5 years (2.33–7.5) *p* = 0.10]. There was a significant difference in terms of APPD (*p* < 0.001), parenchymal thickness (*p* = 0.001), DRF (*p* < 0.05), T1/2 (*p* < 0.05) and Tmax (*p* < 0.001) values in the pyeloplasty group when compared to non-operated renal units. Serum creatinine level was higher in operated patients [median 0.4 mg/dl (0.3–0.47) vs. 0.35 mg/dl (0.26–0.41) *p* < 0.05] although no significant difference was found between eGFR measurements (Table 1).

**Table 1:** Comparison of initial parameters between operated and non-operated patients

	Non-pyeloplasty group (*n* = 136RU)	Pyeloplasty group (*n* = 50RU)	*p*
Weight (kg)	4.6 (3.6 6.17)	4.35 (3.55–6)	0.432
Height (cm)	55 (51–60)	54 (50–59)	0.419
USG (days)	54 (30–90)	40.5 (17–85.5)	0.121
Duration of follow-up (years)	3.41 (1.87–5.97)	4.5 (2.33–7.5)	0.108
APPD (mm)	11 (8.57–14)	23 (15.9–31)	< 0.001
APPD (renal unit)	< 10 mm 48 (35.3%)	< 10 mm 5 (10%)	
	10–14 mm 60 (44.1%)	10–14 mm 3 (6%)	
	≥ 15 mm 28 (20.6%)	≥ 15 mm 42 (84%)	
Parenchymal thickness (mm)	8 (5–9)	4.95 (3.42–6.95)	< 0.05
MAG-3(years)	0.33 (0.25–0.91)	0.25 (0.16–0.5)	0.071
DRF(%)	52 (49–54)	50 (42.5–53)	< 0.05
T _1/2_ (minute)	13.92 (8.25–17.12)	18 (12.75–28.73)	< 0.05
T _max_(minute)	2.48 (2–7.25)	6.48 (3–16)	< 0.001
Renogram curve*	Obstructive 14 (20.9%) Intermediate 19 (28.4%) Non-obstructive 34 (50.7%)	Obstructive 38 (84.4%) Intermediate 5 (11.1%) Non-obstructive 2 (4.4%)	
Serum creatinine (mg/dl)	0.35 (0.26–0.41)	0.4 (0.3–0.47)	< 0.05
eGFR (ml/min/1.73m^2^)	73.5 (57.07–102.54)	68.53 (50.15–108.1)	0.441

Values are expressed as median (interquartile range)

*USG* ultrasonography*, APPD* anterior posterior pelvic diameter*, RU* renal unit, *MAG-3* Mercaptoacetyltriglycine-3, *DRF* differential renal function, *eGFR* estimated glomerular filtration rate

^*^67 renal units in non-pyeloplasty group vs. 45 renal units in pyeloplasty group

*p* < 0.05 indicated statistical significance

According to the results of final ultrasounds and nuclear renograms, renal units underwent surgical procedure had been shown to have significantly elevated median APPD (*p* < 0.001), T1/2 (*p* < 0.05), Tmax (*p* < 0.05) values with decreased cortical thickness (*p* < 0.001) when compared to non-surgical units (Table 2).

**Table 2:** Comparison of final parameters between operated and non-operated patients

	Non-pyeloplasty group (*n* = 113RU)	Pyeloplasty group (*n* = 50RU)	*p*
USG (years)	3.33 (1.72–6)	1.08 (0.58–3.31)	< 0.001
APPD (mm)	8 (6–11)	24 (20–33)	< 0.001
Parenchymal thickness (mm)	9 (811.7)	4.5 (3–6.2)	< 0.001
MAG-3(years)	3.5 (1–4.68)	1.25 (0.75–3.37)	0.064
DRF(%)	50.1 (48–53)	47 (33–53)	0.082
T _1/2_ (minute)	10 (7.5–14)	50.86 (26–79)	< 0.05
T _max_ (minute)	2 (2–4)	4 (2–18)	< 0.05
Renogram curve*	Obstructive 5 (19.2%)	Obstructive 19 (100%)	
	Intermediate 5 (19.2%)	Intermediate 0 (0%)	
	Non-obstructive 16 (61.5%)	Non-obstructive 0 (0%)	

Values are expressed as median (interquartile range)

*USG* ultrasonography*, APPD* anterior posterior pelvic diameter*, RU* renal unit, *MAG-3* Mercaptoacetyltriglycine-3, *DRF* differential renal function

^*^26 renal units in non-pyeloplasty group vs. 19 renal units in pyeloplasty group

*p* < 0.05 indicated statistical significance

### Predictors for intervention

Multivariate regression analysis revealed that initial DRF, T1/2, Tmax, SFU grade 3–4 hydronephrosis, parenchymal thickness and baseline serum creatinine level were not associated with the need for surgery. However, APPD (*p* < 0.05; 95% CI 1.079–1.775) and renogram curve patterns (*p* < 0.05; 95%CI 0.000–0.189) were significant independent risk factors for pyeloplasty (Table 3).

**Table 3:** Multivariate analysis of initial assessments associated with pyeloplasty

Variable	95% Confidence Interval	*p*
APPD	1.079–1.775	0.011
Parenchymal thickness	0.1–10.13	0.52
DRF	0.89–1.17	0.749
Renogram curve	0.000–0.189	0.005
T_1/2_	0.94–1.006	0.103
T_max_	0.97–1.19	0.153

*APPD* anterior posterior pelvic diameter*, DRF* differential renal function,* p* < 0.05 indicated statistical significance

### Outcomes of non-interventional group

**Fig. 1:**
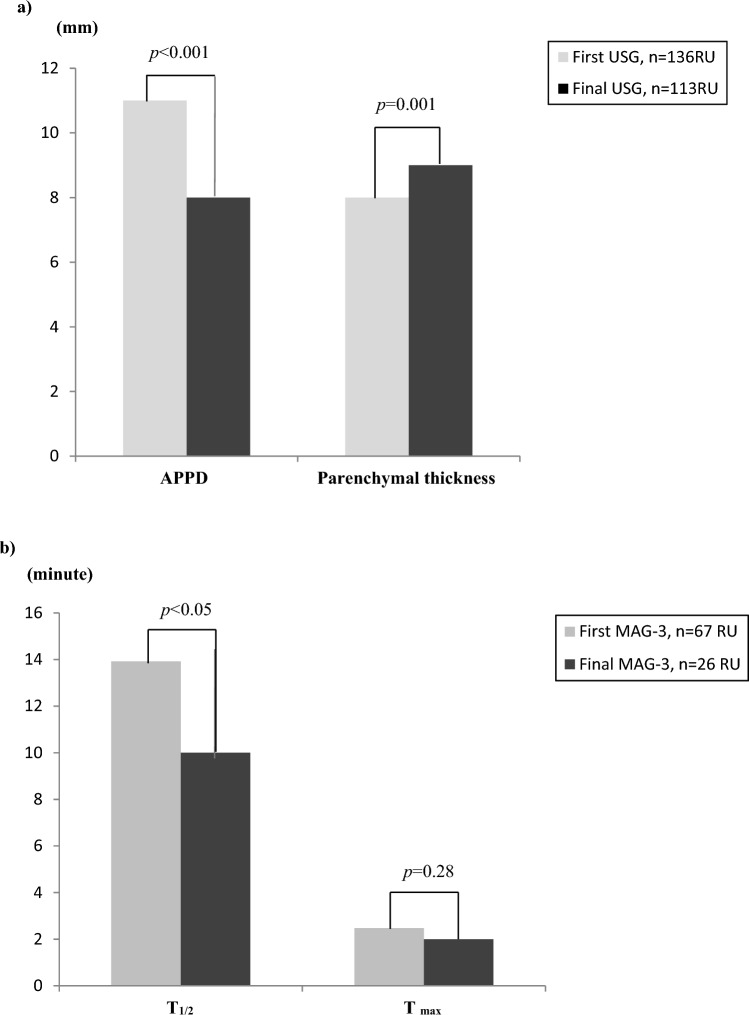
Changes in anterior posterior pelvic diameter *(APPD)*, parenchymal thickness and mercaptoacetyltriglycine-3 (*MAG-3)* parameters in non-operated renal units *(RU)* during follow-up. *p* < 0.05 is significant

In patients who were managed conservatively; a significant decrease in APPD [median 11 mm (8.57–14) vs. 8 mm (6–11) *p* < 0.001) and in T1/2 values [median 13.92 min (8.25–17.12) vs. 10 min (7.5–14) *p* < 0.05] and also significant improvement in parenchymal thickness [median 8 mm (5–9) vs. 9 mm (8–11.7) *p* = 0.001] were demonstrated during the observation period (Fig. 1).

## Discussion

**Fig. 2:**
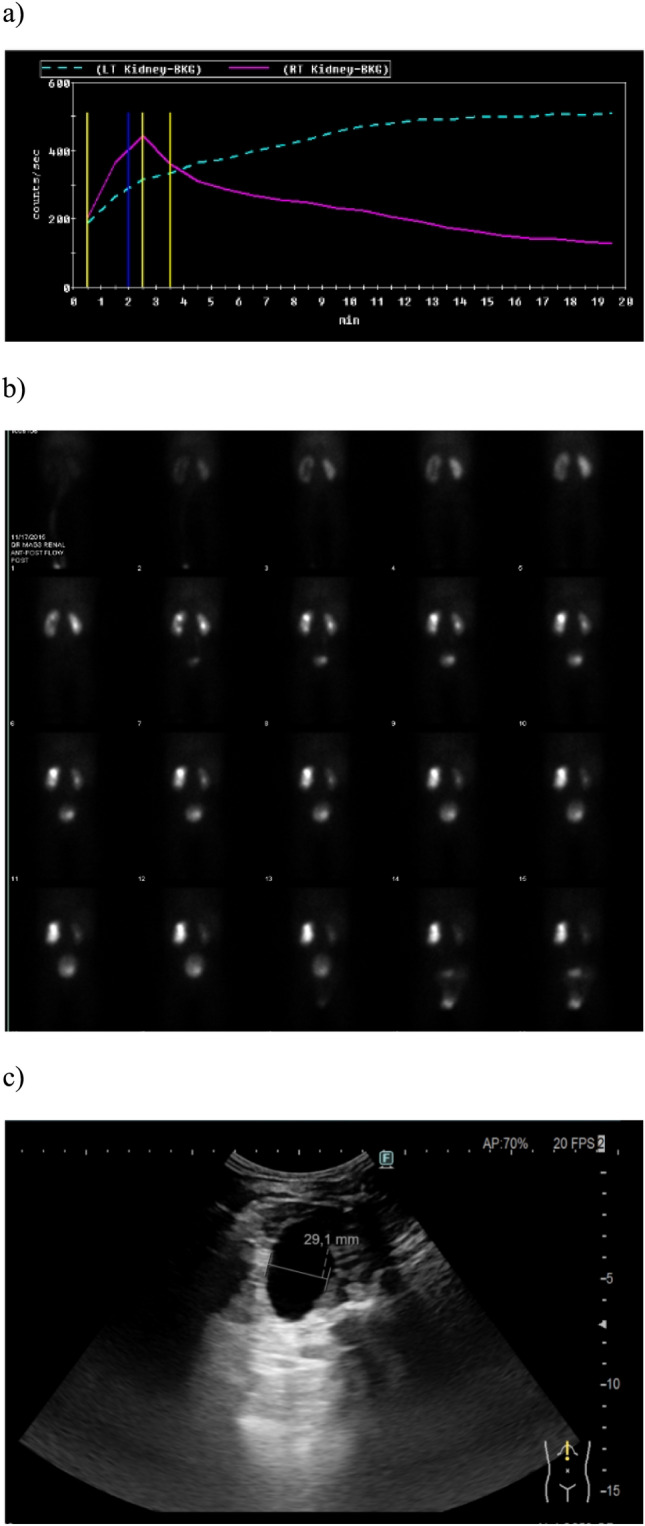
**a, b** Mercaptoacetyltriglycine-3 (*MAG-3*) scan and **c** ultrasonographic images of anteroposterior pelvic diameter (*APPD*) measurement on transverse axis in a patient with ureteropelvic junction obstruction (*UPJO*)

UPJO had been reported to develop approximately one in 1000–2000 live births in the literature with a male predominance. The most common aetiology was reported as an adynamic stenotic ureter segment leading to intrinsic obstruction at ureteropelvic junction [13]. Different parameters have been used before as surgical indications [14]. In a study by Arora et al. [15]; 23.9% of 84 patients with ANH and UPJO have undergone surgical intervention if they had initial DRF < 35%, obstructive curve on renogram or cortical thinning (< 3.5 mm). Patients with an increased SFU grade or APPD, deterioration of renal functions > 5% or > 10% and persistent obstructive patterns on repeated diuretic radionuclide scans during conservative treatment have also been proposed as significant indications for surgery [5, 16]. However, management of UPJO had changed in recent decades. Although the current trend does not recommend surgery immediately after the diagnosis of UPJO because of a probable spontaneous resolution, close monitoring of these patients gained importance to predict worsening functions of the affected kidney. However, there is still controversy on which imaging methods should be preferred and when a patient with UPJO should be operated before permanent kidney damage develops (Fig. 2).

Longpre et al. [16] reported that 24 out of 100 children with UPJO had required surgical correction and initial APPD revealed as an independent risk factor for pyeloplasty. In addition, previous reports had stated SFU grade 3–4 hydronephrosis and DRF less than 40% to be significant predictors for intervention [5]. However, it should be emphasized that multivariate analysis of all these studies were performed on patient populations who had been operated according to the aforementioned indications. However, our work is the first study conducted on patients who underwent pyeloplasty due to UPJO with the definitive diagnosis confirmed both during the surgical procedure and with postoperative histopathological examination.

Due to the evaluation of initial ultrasounds and MAG-3 scans, it was determined that patients who had surgical correction had higher APPD and thinner parenchyme compared to those who did not. Indeed, we demonstrated that APPD and renogram curve pattern were independent risk factors for pyeloplasty; similar with the results of the study highlighting the pelvic diameter measurement in 84 primary UPJO patients [15]. T1/2; which is considered as a sign of poor drainage in hydronephrotic kidney [17, 18]; defines the time it takes for the radionuclide to reach half of its concentration after diuretic injection. Use of T1/2 for the identification of obstruction requiring pyeloplasty is controversial, since it can be influenced by many other factors, such as the degree of hydronephrosis, hydration status, bladder fullness and patient position [19]. In our cohort, while significant difference was present in DRF, T1/2 and Tmax values of operated patients compared to non-operated group; renogram curve pattern; but not radionuclide washout half times, was found to pose the only risk factor for the need of operation. 96 neonates with unilateral UPJO have been monitored by Arena et al. [20] between 1996 and 2006. Out of 66% of 41 newborns who had been demonstrated to have poor drainage (T1/2 > 20 min) on diuretic renograms, spontaneous resolution of hydronephrosis was observed during follow-up without any impairment of renal functions. Indeed, it has been noted in many different studies that T1/2 could not be accepted as a reliable and sensitive tool for the detection of obstructive dilatation [21, 22].

While more than half of antenatally detected pelvicalyceal dilatations resolve during the postnatal period; another 40–45% may stabilize or improve within the first 3 years of life [17, 23–25]. Therefore, a small portion of these children require surgical correction for obstructive hydronephrosis. In the present study, 50 (32.2%) out of 155 patients underwent pyeloplasty and UPJO diagnosis of all operated patients was proven by pathological examination and during surgical procedure. Nevertheless; the incidence of pyeloplasty was similar to 23.9% and 24%, reported by Arora et al. and Longpre et al., respectively [15, 16].

Even though 2 of 45 renal units had non-obstructive renogram curves according to initial MAG-3 scans; we noticed that they had required surgery in follow-up. Therefore, it is noteworthy to state that infants with ANH and pelvic dilatation should continue to be closely monitored both clinically and radiologically; despite the initially normal ultrasound and scintigraphic findings [26, 27].

The limitations of the study are the retrospective design, subjective measurements of APPD or SFU grade by multiple radiologists and the lack of final ultrasound or MAG-3 scan for each participant. Absence of bladder catheterization in infants may confound DRF results on nuclear renograms as well. Since screening for VUR was not currently suggested for mild–moderate hydronephrosis without urinary tract infection or ureteral dilatation, voiding cystourethrography was not applied for each patient.

## Conclusion

Anteroposterior pelvic diameter and renogram curve pattern on the first ultrasound and renal scan were found to be independent predictive risk factors for surgery in UPJO cases whose diagnosis were confirmed with pathological and surgical findings. Although postnatal regression is expected in the majority of those with pelvic dilatation due to ANH; patients should be followed up regularly to prevent probable kidney damage and to predict the need of surgery.
